# Evaluation of Quadratus Lumborum Block as Part of an Opioid-Free Anaesthesia for Canine Ovariohysterectomy

**DOI:** 10.3390/ani11123424

**Published:** 2021-12-01

**Authors:** Jaime Viscasillas, Sandra Sanchis-Mora, Paula Burillo, Vicente Esteve, Ayla Del Romero, Pilar Lafuente, Jose Ignacio Redondo

**Affiliations:** 1 Departamento Medicina y Cirugía Animal, Facultad de Veterinaria, Universidad Cardenal Herrera-CEU, CEU Universities, 46115 Valencia, Spain; Burcabpau@gmail.com (P.B.); vicente.esteve@uchceu.es (V.E.); ayla.delromero@uchceu.es (A.D.R.); nacho@uchceu.es (J.I.R.); 2 The Royal Veterinary College, Hawkshead Lane, North Mymms, Hatfield AL9 7TA, UK; ssanchismora@rvc.ac.uk; 3 Department of Clinical Science and Services, Universidad Internacional de La Rioja (UNIR), Av. de la Paz, 137, 26006 Logroño, Spain; mariadelpilar.lafuente@unir.net

**Keywords:** canine, quadratus lumborum, regional anaesthesia, ultrasound, ovariohysterectomy

## Abstract

**Simple Summary:**

The aim of this study was to assess an anaesthetic protocol involving the quadratus lumborum block (QLB) for canine ovariohysterectomy. Ten healthy dogs were included. Anaesthetic protocol consisted of medetomidine and meloxicam, induction of anaesthesia with propofol and maintenance with sevoflurane in oxygen/medical air. The QLB was performed at the level of L_1_–L_2_ injecting 0.4 mL kg^−1^ of 0.25% bupivacaine/iohexol per side. Computed Tomography (CT) was performed before and after surgery. Fentanyl was administered as rescue analgesia during surgery. The Short Form of The Glasgow Composite Pain Scale and thermal threshold (TT) at the level of the humerus, T_10_, T_13_ and L_3_ were assessed before premedication and every hour postoperatively. Methadone was given as rescue analgesia when it was needed. CT images showed spreading of a median (range) of 3 (2–5) vertebrae, without differences between preoperative and postoperative images. One out of ten dogs needed intraoperative rescue analgesia. Pain score was less than 4/24 in all the animals until four hours after surgery. TT showed significative increase in all the areas tested apart from the elbow 30 min after the recovery. The QLB provides additional analgesia for canine ovariohysterectomy.

**Abstract:**

Quadratus lumborum block (QLB) is used to provide analgesia for abdominal surgery in humans. The aim of this study was to assess an anaesthetic protocol involving the QLB for canine ovariohysterectomy. Ten dogs were included. Anaesthetic protocol consisted of premedication with IM medetomidine (20 μg kg^−1^) and SC meloxicam (0.1 mg kg^−1^), induction with propofol to effect, and maintenance with sevoflurane in oxygen/medical air. QLB was performed injecting 0.4 mL kg^−1^ of 0.25% bupivacaine/iohexol per side. Computed Tomography (CT) was performed before and after surgery. Fentanyl was administered as rescue analgesia during surgery. The Short Form of The Glasgow Composite Pain Scale and thermal threshold (TT) at the level of the elbow, T_10_, T_13_ and L_3_ were assessed before premedication and every hour postoperatively. Methadone was given as rescue analgesia postoperatively when pain score was >3. A Yuen’s test on trimmed means for dependent samples was used to analyse the data (*p* < 0.05). CT images showed spreading of the contrast/block for a median (range) of 3 (2–5) vertebrae, without differences between preoperative and postoperative images. One dog needed rescue analgesia during surgery. Pain score was less than 4/24 in all the animals during the first 4 h after surgery. TT showed a significant increased signal in all the areas tested, apart from the humerus, 30 min after surgery. The QLB may provide additional analgesia for canine ovariohysterectomy. Further studies are needed to assess the specific contribution of the QLB in abdominal analgesia.

## 1. Introduction

The ultrasound (US)-guided quadratus lumborum block (QLB) is a regional anaesthesia technique firstly described in human medicine by Blanco et al. [1]. Since then, several clinical studies have been published in the human literature reporting the analgesic effect of this technique for abdominal procedures [2,3,4]. Based on the results of cadaveric studies, the analgesic effect should be restricted to the mid-caudal abdomen, although clinically, it has also been found helpful for cranial abdominal procedures in human beings [5,6].

Several studies have been published in veterinary medicine describing the technique in dogs [7,8,9,10] and cats [11] and assessing the contrast spreading in cadavers. Three studies administered the contrast between the quadratus lumborum and psoas muscles [7,10,11]. In both dogs and cats, it was found to have a similar distribution, staining the ventral branches of the spinal nerves from L_1_ to L_3_ and the sympathetic trunk from T_13_ to L_3_. Another study in dogs [8] administered the contrast lateral to the QL muscle, and it showed a similar distribution of the spinal nerves (from L_1_ to L_3_), but the contrast was found staining the sympathetic trunk at a much higher level (up to T_11_). Finally, Alaman et al. [9] studied the spreading, administering the contrast dorsal to the QL muscle, and they found similar results. Based on these results, it seems clear that the QLB would provide somatic analgesia for the mid-caudal abdomen, although it might provide visceral analgesia to structures in the medium part of the abdomen.

Marhofer et al. [12] conducted a clinical study in which a mixture of local anaesthetics and contrast was administered to compare the distribution of the dye using MRI versus the clinical extension of the block, eliciting some disparity in the different dermatomes. Interestingly, the analgesia provided in the dermatomes was more extensive than the contrast distribution shown in the MRI images. This result might mean that either local anaesthetic and contrast continue spreading over time or that local anaesthetics move more freely between fasciae than the contrast. 

Although there are some publications in the veterinary literature regarding QLB, none of them have been performed on live animals. Furthermore, there is some evidence in the veterinary literature that contrast spreading is more marked in live animals than in cadavers when an epidural injection of a mixture based on bupivacaine and iohexol is carried out [13]. Therefore, the aims of the study presented here are:

1. To evaluate the antinociceptive effect of this anaesthetic protocol based on QLB during ovariohysterectomy in dogs;
2. To assess the analgesic effect of the QLB during the postoperative period;
3. To compare the extension of the contrast’s spreading after it was administered in the QLB before and after surgery;
4. To evaluate the complications related to the QLB during surgery and the postoperative period.

We hypothesise that this anaesthetic protocol based on the QLB will avoid nociceptive response during ovariohysterectomy and provide analgesia during the postoperative period. We also hypothesise that the spreading of contrast will be more extensive after surgery, and no severe side effects will be found with this technique.

## 2. Materials and Methods

This study was undertaken at the Universidad CEU-Cardenal Herrera in Valencia (Spain) after obtaining ethical approval from the University Ethical Committee (CEU-001-2018). Ten female dogs of different ages, body conditions and breeds undergoing ovariohysterectomy were included in the study. A pre-anaesthetic evaluation was carried out in every dog, including a physical examination, haematology, biochemistry, ECG and two orthogonal thoracic radiographs. The inclusion criteria were female dogs, undergoing elective ovariohysterectomy and having an ASA-I status. The exclusion criteria were nervous or aggressive behaviour, ASA ≥ II or dogs with previous abdominal surgery. 

**Figure 1:**
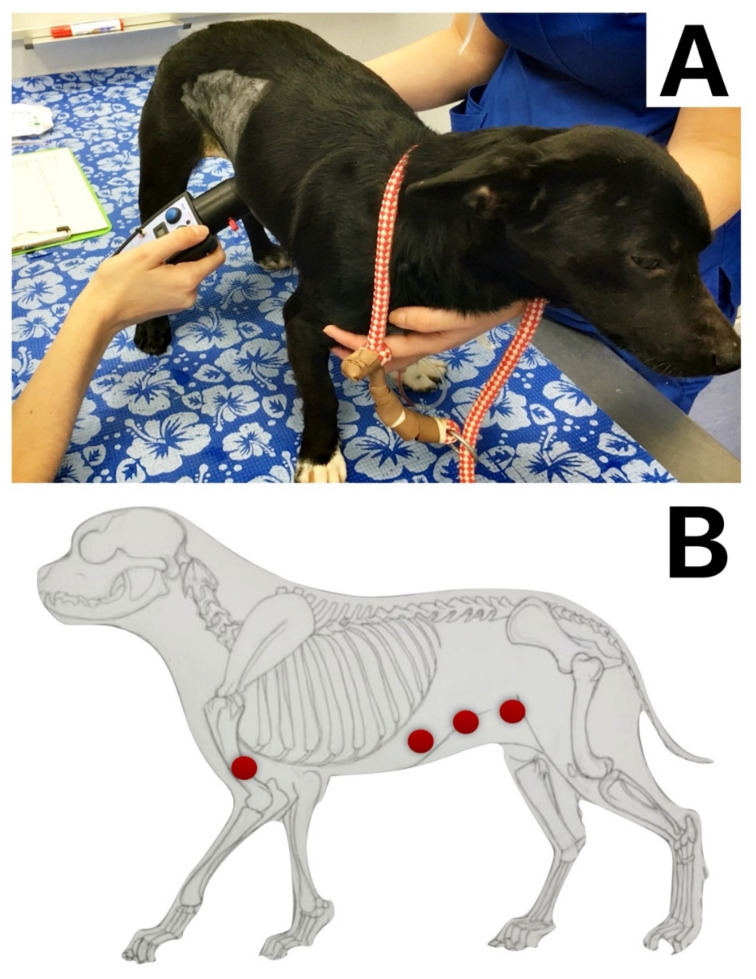
(**A**). Picture showing the thermal threshold (TT) assessment in a dog after surgery. (**B**). Red bullets showing the body areas where the TT was assessed in each animal. They represent the left lateral humerus area and bilaterally at the T10, T13 and L3 dermatomes.

A pain assessment was conducted using the Short Form of The Glasgow Composite Pain Scale and the thermal threshold (TT) in all dogs one day before surgery to obtain basal values. The TT was performed at the level of the humerus (as a control area) and the skin of the abdominal wall, in dermatomes T_10_, T_13_ and L_3_. Heat stimulus was applied using a veterinary thermal probe (HotPro; Topcat Metrology Ltd., Ely, UK) using a protocol described by Sanchis-Mora et al. [14]. The hair of the different body areas (BAs) was clipped (Figure 1). Before testing, the skin temperature was measured with the device, and the room temperature was recorded (EL-USB-TP-LCD; Lascar Electronics, Salisbury, UK). During testing, the device temperature was increased from baseline to a maximum of 55 °C with a ramp of 1 °C s^−1^ until the endpoint was reached. The endpoint was defined as a movement of the animal reacting to the temperature or when device temperature reached its maximum. It was repeated 3 times in each of the seven BAs. Twenty seconds were allowed between repetitions. The final threshold of the test per BA was obtained by calculating the mean of the three repetitions per BA. The order of assessment of the BAs was randomly allocated using the software https://www.random.org (accessed on 6 August 2021).

The day of the surgery, dogs were premedicated with an intramuscular (IM) medetomidine at 20 mcg kg^−1^ and subcutaneous (SC) meloxicam at 0.2 mg kg^−1^. Once the animals were sedated, induction of anaesthesia was achieved with intravenous propofol. Once the endotracheal tube was in place, sevoflurane in a 50:50 mixture of oxygen and medical air was administered through a circular breathing system. The expiratory fraction of sevoflurane was set up at 1.8% in all the animals.

Once the dogs were monitored and stable, they were placed in lateral recumbency to perform the ultrasound-guided QLB technique.

The hair in the flank was clipped, and the skin was aseptically prepared with chlorhexidine. The blocking technique was carried out twice in each animal, with one injection per side. The same anaesthetist performed the blocks using a 22-gauge, 88 mm or 22-gauge, 35 mm Quincke spinal needle (Spinocan; B. Braun, Recklinghausen, Germany). The anaesthetist chose the size of the needle based on each dog’s size, body condition, and personal experience. A linear 13-6 MHz ultrasound probe (Sonosite HFL38/13-6 Ultrasound Probe; Sonosite, WA, USA) attached to an ultrasound machine (X-Porte, Sonosite, WA, USA) was used in all the animals. The needle was connected to an extension set (Discofix C; B. Braun) and a syringe (Omnifix; B. Braun). A total volume of 0.4 mL kg^−1^ of a 50:50 mixture of iomeprol (Iomeron; Bracco Imaging, Milano, Italy) and bupivacaine 0.5% (Bupivacaine 0.5%, B. Braun, Recklinghausen, Germany) was administered at each injection site. 

The US-guided QLB technique was performed in the following manner. The US probe was placed in a transverse plane over the lumbar muscles just caudal to the last rib using a paramedian sagittal oblique (subcostal) approach described by Viscasillas and colleagues [10] in dogs. Once in position, the probe was slightly rotated cranially to visualise the following anatomical structures: quadratus lumborum muscle, psoas muscle, lateral aspect of the body of the first lumbar vertebrae and the transverse process of the first lumbar vertebrae. The needle was inserted in-plane and advanced at a 45° angle from dorsolateral to ventromedial until its tip was placed between the quadratus lumborum and psoas muscles. At this location, the mixture was injected into the fascial plane. A volume of less than 0.5 mL of saline was first injected to confirm the correct position of the tip of the needle. The injection was considered valid when hydrodissection of the quadratus and psoas muscle, together with the ventral movement of the anterior thoracolumbar fascia, were noted. If this pattern was not visualised, the needle was redirected until this hydrodissection was observed. Once the correct position was confirmed, the entire volume of the local anaesthetic/contrast mixture was administered.

The CT images were taken two times, five minutes after bilateral QL blocks were performed and at the end of surgery. The scanner employed was a Brivo CT 385 (GE Hangwei Medical Systems Co., Ltd., Beijing, China) set at 120 kV, 199 mA and 1.25 mm slide thickness parameters. A 3D reconstruction of the CT images was performed using Horos DICOM Medical Image Viewer (Horos Project, 2018), and a veterinary radiologist assessed the images. The parameters evaluated in the CT images were the extension of contrast distribution based on the number of vertebrae.

After obtaining the first CT images, the dogs were moved to the theatre, where another investigator, different from the previous and unaware of the CT results, administered and managed the anaesthesia during the surgical procedure. The same surgeon performed all the ovariohysterectomies and was also unaware of the CT results. Animals were monitored using a multiparametric monitor (Carescape B650, GE Healthcare, Boston, MA, USA). Parameters recorded included ECG, end-tidal carbon dioxide, arterial haemoglobin saturation, the expired fraction of sevoflurane, temperature, tidal volume, compliance, cranial reflexes and non-invasive blood pressure (oscillometric method). All animals were mechanically ventilated using a volume control mode to maintain end-tidal carbon between 35 and 45 mmHg, with a maximum peak inspiratory pressure (PIP) of 15 cm H_2_O and I:E of 1:3. All the parameters (heart rate, blood pressure, respiratory rate and expired fraction of sevoflurane) were recorded just before the first incision. Any increase of more than 20% from baseline heart rate and blood pressure values and/or dyssynchrony related to any surgical stimulus was considered as nociception and rescue analgesia was administered. Intravenous fentanyl at 5 mcg kg^−1^ was used as rescue analgesia every time it was needed. Hypotension was defined as mean arterial blood pressure below 60 mmHg.

Once the surgery was finished, the dogs were moved to radiology, where another CT was carried out. Animals were recovered after it. Recovery was defined as the moment when the dog was able to walk without ataxia.

Dogs were assessed for pain using the Short Form of The Glasgow Composite Pain Scale and Thermal Threshold in the same BAs as previously described. It was performed 30 min after recovery, then 1 h, 2 h, 4 h and then, every 4 h until the animal was discharged from the hospital. Intravenous methadone at 0.2 mg kg^−1^ was administered as rescue analgesia if the pain score was higher than 4 during the postoperative period. The time of the rescue analgesic administration was recorded, and the dogs were removed from the study at that point. Postoperative follow-up was continued as routine until they were discharged from the hospital.

**Figure 2:**
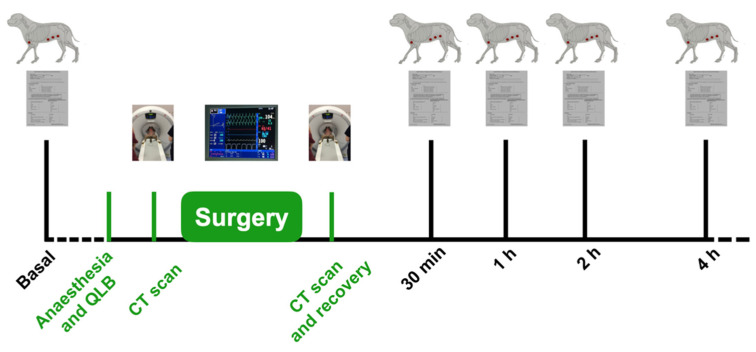
Timeline of the experiment. Basal values of TT and pain score were taken one day before the ovariohysterectomy. The day of the surgery, the dogs were anaesthetized and the QLB was carried out. CT scans were performed before and just after surgery. Pain score and TT values were recorded 30 min after recovery and 1, 2 and 4 h after recovery.

Parameters analysed include pain score values before and after surgery, TT values before and after surgery in the described BAs, rescue analgesia needed during surgery, evaluation of contrast spreading based on CT images before and after surgery and complications related to the QLB (Figure 2).

The results were analysed using R 4.0, a free software environment for statistical computing and graphics (R Core Team, 20). They are displayed as median (range). A Yuen’s test on trimmed means for dependent samples was used to analyse the data. A *p*-value < 0.05 was considered statistically significant. 

## 3. Results

Ten dogs were included in the study. They were all crossbreed, entirely female, ASA I, weighing 15.7 (4.2–27) kg, a body condition score of 5 (4–8) based on the AAHA nutritional assessment guidelines for dogs and cats [15] and being 22 (8–56) months of age.

Time from sedation to recovery was 150 (120–190) min and from the QLB to recovery 90 (70–120) min.

**Figure 3:**
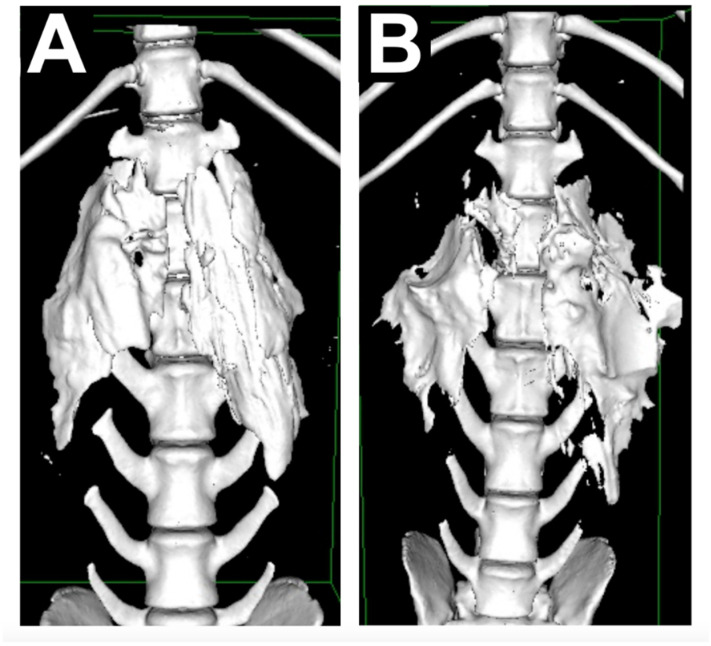
(**A**) CT image showing the contrast distribution in a dog just before surgery. (**B**) CT image of the same animal after surgery.

The CT images showed the contrast spreading a median of 3 (2–5) vertebrae before surgery and 3 (2–6) vertebrae after surgery. No statistical difference was found between them (*p* = 0.98) (Figure 3).

Rescue analgesia was administered in one animal (1/10) during the surgical procedure. All the baseline values (heart rate, respiratory rate and blood pressure) increased more than 20%. It was needed during the manipulation of the first ovarian pedicle. However, no more rescue analgesia was necessary for that animal. No changes in the baseline values during surgery were recorded apart from the dog who received rescue analgesia

**Figure 4:**
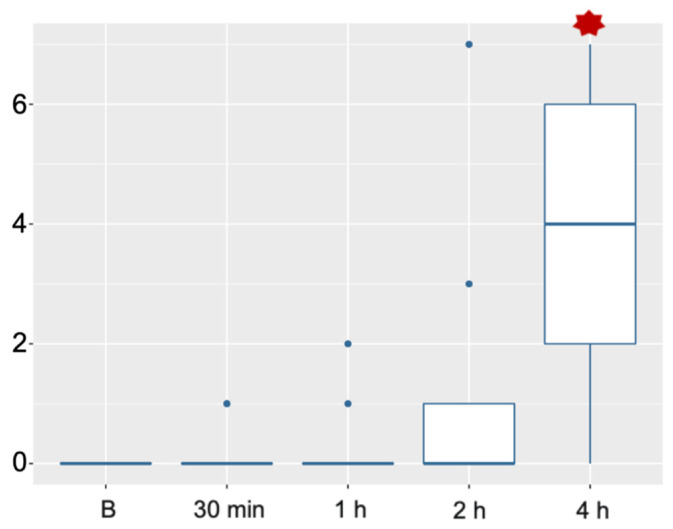
Pain score values taken the day before surgery (B) and 30 min, 1, 2 and 4 h after surgery. Statistical difference was just found 4 h after surgery, where the median of pain score values was 4.

Postoperative pain score values did not show statistical differences from the values recorded before surgery, 30 min and 1 h after recovery. Although values started to increase 2 h after surgery, they were not statistically different. Statistical difference was found 4 h after surgery, where nearly all the animals needed rescue analgesia (Figure 4).

**Figure 5:**
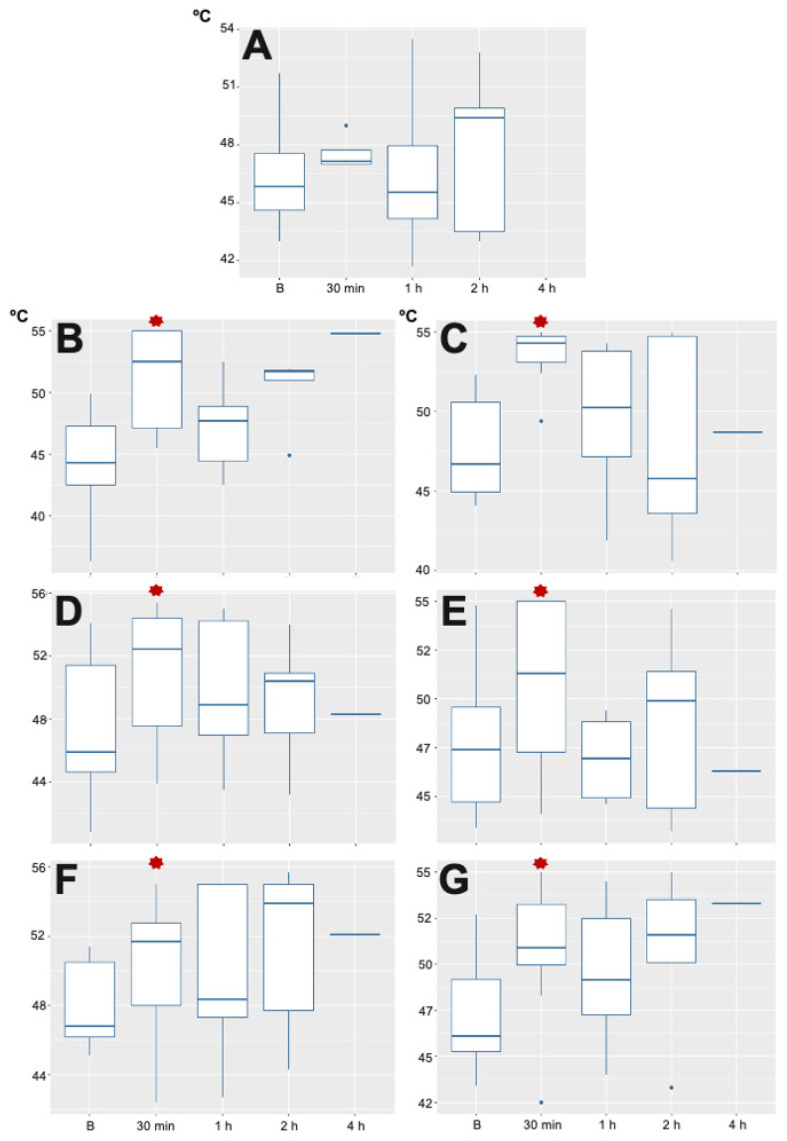
Thermal Threshold results found in the different body areas. (**A**) humerus (control body area), where no differences were found at any time; (**B**) (left T10 dermatome); (**C**) (right T10 dermatome); (**D**) (left T13 dermatome); (**E**) (right T13 dermatome); (**F**) (left L3 dermatome) and (**G**) (right L3 dermatome) show statistical difference just 30 min after surgery.

Regarding the TT, there were no statistical differences at any time at the level of the mid humerus. There was a statistical difference in T10, T13 and L3 dermatomes 30 min after recovery on both sides of the abdomen compared with basal values. However, no statistical differences were found later on in any area (Figure 5). The TT assessment was carried out just in one dog 4 h after the recovery. The rest of the dogs were reacting as soon as the TT assessment was started and no confident values were collected, therefore they were not included in the analysis.

Finally, no complications were recorded during surgery, such as hypotension or bleeding, or the postoperative period, such as vomiting or ataxia.

## 4. Discussion

In dogs undergoing ovariohysterectomy, QLB performed by administering bupivacaine 0.25% at 0.4 mL kg^−1^ per side allowed a satisfactory intraoperative analgesic plan in a relatively light anaesthetic plane (ETSevo about 1.8%). Furthermore, the QLB as described in this study provided an adequate postoperative analgesia for up to 4 h after recovery. Only one animal needed rescue analgesia during surgery, which occurred during ovarian ligament manipulation, one of the most recognised critical points during ovariohysterectomy [16]. This animal did not react during the rest of the surgical procedure, so we can assume that the block was incomplete in all areas.

Ovariohysterectomy has been used in dogs as a model for somatic and visceral pain [16]. Our results support the hypothesis that the QLB would cover both components. The preoperative spreading of contrast found in our study was similar to the results reported in previous cadaveric studies that used the same injection site [7,10] although administered volume was higher in our study (0.15 to 0.3 vs. 0.4 mL kg^−1^ respectively). Accordingly, the spreading of contrast is similar in live animals and thawed cadavers [7,10]. The results of our study support the hypothesis that the quadratus lumborum block would provide adequate analgesia for this surgery. Considering the distribution, which covers from T13 to L3, the QLB is adequate for ovariohysterectomy, as the abdominal wall innervation and skin [17] are mainly covered for those nerve roots.

With regard to visceral analgesia for abdominal organs, ovarian tissues and uterus, it is provided by sympathetic fibres. Paravertebral ganglia is responsible for the sympathetic innervation of the ovarian tissues and receives inputs from T_11_ to L_4_ segments, with the majority located between T_13_ and L_2_ [18]. Uterus sympathetic innervation arises from the hypogastric nerve, which originates in L_1_ to L_4_ segments of the spinal cord [18]. These areas were found to have been stained by the contrast injected during the QLB in all the dogs.

Another significant result in our study, based on the CT images taken, was that the spreading pattern of the contrast media did not increase after surgery. Therefore, it is unlikely that the pattern of distribution increases with time. The same conclusions were raised in some human studies [1], although others found further spreading 2 h after the injection [19]. However, there are some potential limitations to our results. CT images were taken 5 min after the QLB was carried out and immediately after surgery, which could be a slightly different time among animals. Additionally, no CT images were taken at other fixed times between the first CT scan and the last one. Studies carried out in human medicine performed CT or MRI images nearly every hour [19,20]. Therefore, it is uncertain if the spreading of contrast could have increased over time and have been partially reabsorbed later on before the postoperative CT scan was performed. However, after studying the CT images, no trace of contrast could be observed, as it could have potentially distributed further and have been reabsorbed.

Furthermore, based on the contrast spreading observed in the CT images, we did not expect to reach a blockade at the level of T_10_. However, we found a significant difference in the thermal threshold at this level 30 min after the end of the surgery. The local anaesthetic might reach T_10_ even if the radiological contrast does not show distribution at that level. Our results agree with Marhofer et al. [12], who found that the distribution of radiological contrast did not correlate with the distribution of local anaesthetic. Unfortunately, this fact cannot be assessed in our study with the methodology used. The Short Form of The Glasgow Composite Pain Scale used in our study has been previously validated to evaluate acute pain in dogs, and it is widely used in clinical studies [21,22]. Although a pain score higher than 5/24 has been reported as the cutoff point to administer rescue analgesia, we decided to use a value of 4/24. This decision was based on our previous clinical experience where dogs seem to have very low pain scores during the recovery period, but these seem to increase over time once the local anaesthetic starts wearing off. Furthermore, the NSAIDs were administered preemptively to prevent the establishment of the inflammatory component of the acute pain, which has contributed to the overall success of the analgesic plane. Our results show that pain scores were low after recovery, but there was an increasing trend of the pain scores over time. This finding would be related to the end of the effect of the local anaesthetics. Other studies have shown bupivacaine’s more prolonged clinical effect when being administered in a TAP block in cats [23]. However, the bupivacaine concentration used in our study was lower than in other studies, which could be a potential reason for our results.

The thermal threshold has been used in previous studies to get a more objective measurement of the analgesic effect [24,25]. In dogs, TT has been reliably measured [26] to evaluate pain in different conditions [27,28,29] and to evaluate the efficacy of analgesics [30]. Thermal threshold testing has been used in human anaesthesia to assess the effect of loco-regional anaesthesia techniques and the differences among several drugs [25]. Our study showed a significantly increased thermal threshold in all dermatomes (T_10_, T_13_, _L3_) on both sides of the skin of the abdominal wall when it was assessed 30 min after the recovery, compared with the basal values before the block. All these post-block values were not significantly different among them for the rest of the evaluation period. The thermal threshold remained similar at all times in the elbow area. Therefore, it could be inferred that any systemic drugs administered had little or no effect on the results found in the abdomen. 

Although it was not an aim of the study, it seems that there is a potential correlation between the pain score and TT results: significantly high TT results anticipated low pain scores. However, when TT values decreased, pain scores started to increase. Therefore, TT might be an excellent method to objectively assess analgesia and anticipate when the effect of the local anaesthetic could be wearing off. However, other studies must be conducted on this topic to confirm our hypotheses.

No complications were recorded during this study regarding the QLB. Hypotension has been described in human literature due to extensive sympathetic block [31], but we did not encounter any case with this side effect during anaesthesia or the postoperative period. On the other hand, hind limb weakness has also been reported with this QLB approach [32]. It was not noted in any of our cases. No delay in recovery was recorded in any of their animals, and standing and walking capacity were present in all dogs at first post-operative assessment, 30 min after the end of surgery.

Our study has some limitations. The first limitation is the lack of a control group, which could raise questions about the main analgesic effect being caused by the QLB. However, the results found in all the cadaveric studies published in dogs have shown that the QLB can potentially provide analgesia in these types of cases. The spreading of contrast observed in our study was similar to the cadaveric studies; therefore, it is sensible to think that the intraoperative analgesia was mainly reached by the use of the QLB. On the other hand, there is insufficient evidence from the literature to ensure that intramuscular medetomidine and NSAIDs administration could be a suitable analgesic protocol to provide adequate analgesia for this surgical procedure. Although medetomidine provides analgesia, its analgesic effect has been reported to be half the duration of the sedative effect [33,34]. Therefore, the authors believe the analgesic effect had a minimum impact during surgery and the postoperative period.

Furthermore, the increase in the pain score values once the effect of the bupivacaine started to wear off (based on its theoretical duration of action) makes us believe that the loco-regional technique mainly provided analgesia. Another limitation in this study is that investigators were not blinded to the treatment administered so that the results could be biased. However, only one anaesthetist assessed the intraoperative nociception, and rescue analgesia criteria were previously set up. Two anaesthetists assessed pain during the postoperative period; one carried out the TT test, and the other one the pain scores. Both operators took the data without knowing the other results during the intraoperative phase or those from the other postoperative method. In that way, an effort was made to avoid bias. Finally, although the TT test is an objective evaluation method, it is essential to consider that the interaction between the operator and the dog, the relationship between the response and the perceived intensity of the stimulus in non-verbal species, and the attention and motivation of the dog can all bring variability on TT [35]. The operator who carried out the TT test had previous experience using this device. Despite this, the operator could not carry out all the measurements on T_2_ and T_4_ in all of the dogs. It could have occurred because some dogs were already feeling discomfort at the evaluation time or learned from previous assessments and did not want to collaborate.

## 5. Conclusions

Based on our results, the QLB is an effective and safe alternative to provide analgesia for ovariohysterectomy in the dog. As per previous studies, the spreading of contrast after the QLB seems similar in live dogs than in canine cadavers. No difference in spreading was found over time. The analgesia provided during the postoperative period is adequate, although clinicians should be aware of the duration of the local anaesthetic administered to the animal. Further clinical studies are needed to assess the effect of the QLB under other scenarios.

## Data Availability

Data supporting he reported results can be sent to anyone interested by contacting the corresponding author.
